# Comparison of Newer Hand-Held Ultrasound Devices for Post-Dive Venous gas Emboli Quantification to Standard Echocardiography

**DOI:** 10.3389/fphys.2022.907651

**Published:** 2022-06-09

**Authors:** Kamellia Karimpour, Rhiannon J. Brenner, Grant Z. Dong, Jayne Cleve, Stefanie Martina, Catherine Harris, Gabriel J. Graf, Benjamin J. Kistler, Andrew H. Hoang, Olivia Jackson, Virginie Papadopoulou, Frauke Tillmans

**Affiliations:** ^1^ Joint Department of Biomedical Engineering, The University of North Carolina at Chapel Hill and North Carolina State University, Chapel Hill, NC, United States; ^2^ Divers Alert Network, Durham, NC, United States

**Keywords:** decompression sickness, decompression stress, diving, bubble, Doppler

## Abstract

Decompression sickness (DCS) can result from the growth of bubbles in tissues and blood during or after a reduction in ambient pressure, for example in scuba divers, compressed air workers or astronauts. In scuba diving research, post-dive bubbles are detectable in the venous circulation using ultrasound. These venous gas emboli (VGE) are a marker of decompression stress, and larger amounts of VGE are associated with an increased probability of DCS. VGE are often observed for hours post-dive and differences in their evolution over time have been reported between individuals, but also for the same individual, undergoing a same controlled exposure. Thus, there is a need for small, portable devices with long battery lives to obtain more ultrasonic data in the field to better assess this inter- and intra-subject variability. We compared two new handheld ultrasound devices against a standard device that is currently used to monitor post-dive VGE in the field. We conclude that neither device is currently an adequate replacement for research studies where precise VGE grading is necessary.

## 1 Introduction

There are between 2.7 and 3.5 million active scuba divers in the United States and approximately six million active scuba divers worldwide (Diving Equipment and Marketing Association, 2022). Scuba divers breathe compressed gas supplied at ambient pressure throughout the dive. While oxygen is metabolized, inert gases (nitrogen in case of compressed air) diffuse into the blood and saturate the tissues throughout the dive while ambient pressure is increased and maintained. During ascent, the pressure gradient reverses and accumulated inert gas in tissues is released back into the circulation and breathed out. Gas is eliminated as dissolved gas in plasma but may also accumulate to form small bubbles (free gas phase) around micronucleation sites in supersaturated tissues or blood (Papadopoulou et al., 2013). Bubbles in circulation are referred to as venous gas emboli (VGE) when detected with ultrasound, where they can be seen flowing towards the lungs where they are normally dissolved/filtered out by diffusion. In rare cases, these VGE may cross from the venous to the arterial circulation through right-to-left shunts, such as a patent foramen ovale (PFO) or lung shunts (Mitchell et al., 2022) (Papadopoulou et al., 2014). High amounts of post-dive VGE are associated with greater risk of developing decompression sickness (DCS) (Nishi et al., 2003) (Eftedal et al., 2007).

Decompression sickness is a risk not only associated with scuba diving but can also occur in other instances of reduced ambient pressure, including during compressed air work, high altitude, extra-vehicular activity in space, or unpressurized air travel. Symptoms of DCS range from mild to severe, from skin irritations, fatigue, nausea and dizziness to stroke-like symptoms, muscle weakness, paralysis or death. The standard treatment for DCS is timely administration of oxygen to accelerate inert gas diffusion and increase partial pressure of oxygen (pO_2_) to tissues, followed by recompression in a hyperbaric chamber, and adequate fluid management (Chin et al., 2017). Inadequate or delayed treatment may result in lasting damage through mechanical disruption of neural tissue by inert gas emboli, hypoxemia through impaired perfusion, or activation of inflammatory pathways (Mitchell et al., 2022) (Newton, 2001).

**FIGURE 1:**
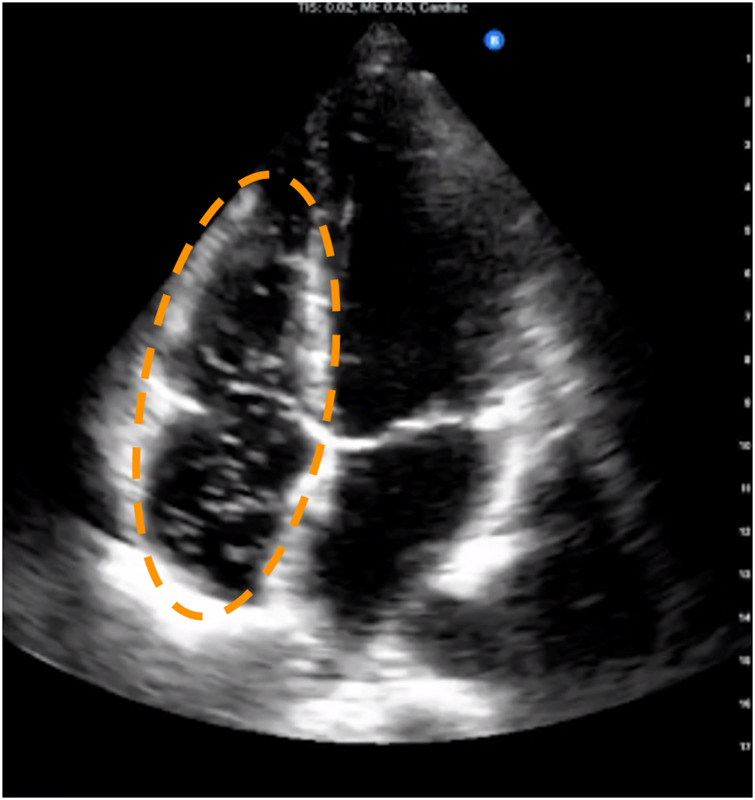
Example frame from four-chamber trans-thoracic echocardiography showing venous gas emboli circulating in the right atrium and ventricle. These appear as bright spots against the dark background that is the blood inside the chambers. The dashed ellipse outlines the venous chambers where venous gas emboli are typically seen when present.

Current standard equipment for monitoring VGE in the field are laptop-style clinical 2-dimensional (2D) ultrasound devices (Le et al., 2021). Visualization yields echocardiograms which show the VGE circulating the heart chambers (Figure 1). VGE in 2D echocardiography recordings are quantified using the Eftedal-Brubakk (EB) scale–a 0–5 grading scale where 0 represents no bubbles, and 5 represents a “wash-out” where single bubbles cannot be discriminated. The risk of developing DCS post-dive increases at EB grades greater than or equal to 3 (Eftedal et al., 2007).

Doppler ultrasound is another established way to monitor the presence of post-dive VGE by collecting audio recordings of moving VGE in blood. Doppler recordings use one of two positions on the diver’s thorax to assess VGE presence: precordial or subclavian. Doppler ultrasound quantification is performed on the Kisman Masurel (KM) scale, which quantifies bubbles using frequency, loudness, and duration of bubble signals (Kisman and Masurel, 1983), or using Spencer grading (Spencer and Johanson, 1974) (Nishi et al., 2003).

While a high presence of post-dive VGE, whether measured with Doppler or 2D echocardiography, is linked to higher rates of DCS, the mere presence of VGE does not lead to DCS (Nishi et al., 2003). In fact, the amount of VGE after diving varies significantly between individuals even after completing identical, controlled dives (Papadopoulou et al., 2018). Explaining this variability is a major goal toward personalizing decompression procedures and guidelines for individual divers. While certain risk factors for DCS, like presence of a right-to-left shunt, age, weight, or sex, cannot be controlled, it is important to be able to understand why some individuals experience more incidents of DCS and/or VGE than others even after completing identical dives. Studies on inter- and intra-subject variability require many volunteers, repeated dives per volunteer and numerous recordings to capture the evolution of VGE for hours post-dive. Since many field experiments are performed on small boats or in remote places where large, bulky devices that require stable electric power prove inconvenient, portability and long battery life are two desirable attributes in ultrasound devices. Thus, the focus of this study was to determine if two new and more portable systems could yield similar VGE classification as our standard device and therefore be used in the field to gather meaningful data for future physiological studies to contribute to new diving safety standards.

## 2 Methods

Three devices were compared in this study. As the current standard for circulating VGE-related research, a laptop-style 2D ultrasound device, the Vivid q™ (GE Healthcare, Chicago, IL) was used. For comparison, a handheld portable 2D-ultrasound probe connected to a commercially available tablet, the Butterfly iQ™ (Butterfly Network, Guilford, CT), and a handheld subclavian Doppler device with wireless connection to a tablet, the O’Dive™ (Azoth Systems, Ollioules, France) were used. The study received ethics approval by the institutional review board of the Divers Alert Network and all procedures and methods were implemented accordingly.

### 2.1 Study Population

All volunteers were informed of the protocol for the study and consented prior to participation. To be eligible, volunteers had to be at least 18 years of age and be certified to a minimum level of “Advanced Open Water Diver” with 50 logged lifetime dives. A total of 75 scuba divers were recruited at pre-determined monitoring sites over 21 months and underwent pre- and post-dive VGE monitoring. Divers were unrestricted in preparing for and conducting their dives, so that the dive profile (time and depth underwater) was not dictated.

### 2.2 Ultrasound Acquisitions

Volunteers were measured with ultrasound before their dive, as a baseline, and at set times after their dive. It was expected that not every volunteer would present with VGE, however, if VGE would be observed, this highly dynamic process would demand fast and efficient measurements with each device shortly after the other to ensure the results for set time points could be compared (Blogg and Gennser, 2011) (Møllerløkken et al., 2016) (Papadopoulou et al., 2018). The order of the Butterfly iQ™ and O’Dive™ measurements was therefore alternated for each volunteer post-dive, with the Vivid q™ always being the second device used on the volunteer.

**FIGURE 2:**
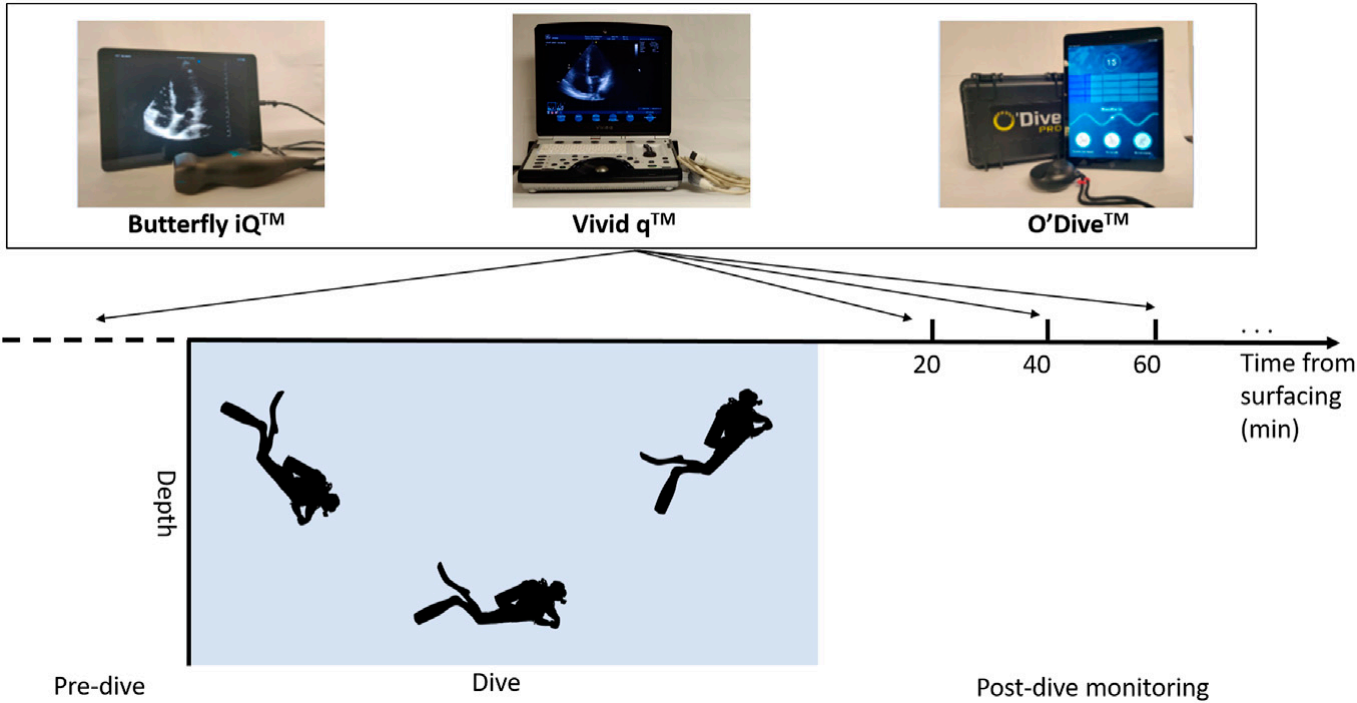
Schematic of experimental protocol depicting pre- and post-dive ultrasound recording time points. All three devices were used at each time point with the Vivid q™ being the second measurement.

Post-dive measurements occurred approximately at 20, 40, and 60 min after the dive. Additional measurements were taken at 20-min intervals if volunteers still had VGE by the end of the 60-min initial monitoring period. At each time point, measurements were taken with three separate devices, as depicted in Figure 2. The current standard for VGE imaging in diving field experiments is B-mode echocardiography on laptop-style portable devices such as the Vivid q™ (GE Healthcare, Chicago, IL). This system has been used in numerous studies by the Divers Alert Network research foundation (Zanchi et al., 2014) (Thom et al., 2012), and others (Šegrt Ribičić et al., 2019), in the past and was used as the benchmark for comparing newer hand-held devices. The two hand-held devices assessed for their agreement in VGE quantification with the standard Vivid q™ were the Butterfly iQ™ (Butterfly Network, Guilford, CT) and O’Dive™ (Azoth Systems, Ollioules, France).

The Butterfly iQ™ ultrasound probes are designed to be used as an easily portable, point-of-care ultrasound (POCUS) device compatible with common commercial mobile devices. Once the user has downloaded and started the Butterfly IQ™ application (app) on their desired device, the Butterfly iQ™ probe can be plugged into that device and the app’s user interface will display the mechanical index (MI) and thermal index (TI) of the probe at the top of the screen. A toolbar at the bottom of the app’s user interface will allow the user to select predefined sets of imaging parameter values, enter image capture mode, select tools, and freeze/unfreeze any image already on screen. When an ultrasound image is displayed, the option to capture images and record clips will appear, after which they may be uploaded to a “capture reel” and saved to the device or the Butterfly Network™ cloud.

During transthoracic echocardiography acquisitions with the Vivid q™ and Butterfly iQ™, volunteers were lying on their left side and the probe was placed on the fourth or fifth intercostal space to obtain an apical four-chamber view. The standard view was modified by rotating the probe slightly ventrally so the right atrium and ventricle could be completely viewed as per Germonpré et al. (2014). Recordings consisted of at least 10 cardiac cycles and videos were stored locally on the device until they could be transferred to a HIPAA-compliant cloud.

The O’Dive™ is a new device marketed to scuba divers and the first of its kind providing Doppler ultrasonic self-monitoring as a possible mean to improve diver safety. Once the O’Dive has been registered and connected to a compatible device with the O’Dive™ app, it can be used by divers after surfacing to record VGE via continuous wave Doppler by placing it under the left and right clavicle for approximately 20 s each. To take a measurement, an identifier must be input into the app before the operator selects “new measurement”. This will prompt the user to ensure their sensor is properly paired before receiving instructions on how and where to place the sensor. During the measurement, the user is instructed to remain still and breathe in time with the prompts. The system then gives a score of measurement accuracy. Should the placement be incorrect, or the measurement perceived as not satisfactory, the user will be asked to attempt the measurement again. In our study, the O-Dive™ was positioned on the subclavian vein on each side of the body to obtain measurements as the volunteer sat upright, in accordance with its intended positioning. Instead of having each volunteer self-monitor, recordings were taken by experimenters since the study aims to assess the device for possible research use in field experiment conditions (not self-monitoring).

### 2.3 Echocardiography and Doppler Venous gas Emboli Assessment

The 2D echocardiograms obtained by the Vivid q™ and Butterfly iQ™ were graded using the EB scale (defined in Table 1) by two independent raters (KK and AH), and when their values disagreed, an additional independent rater (VP or FT) also reviewed them and reached consensus with all raters. VGE data obtained with each device was also binarized based on the presence (or lack) of bubbles. The Vivid q™ data was used as a standard for comparison with the Butterfly iQ™ and O’Dive™, respectively.

**TABLE 1:** Definition of the Eftedal and Brubakk venous gas emboli (VGE) grading used in echocardiography analysis, adapted from (Eftedal et al., 2007). The third column shows the binary classification based on VGE presence.

Grade	Detailed description	VGE present
0	No observable bubbles	No
1	Occasional bubbles	Yes
2	At least one bubble every four cardiac cycles	Yes
3	At least one bubble every cardiac cycle	Yes
4	At least one bubble per cm^2^ in every image	Yes
5	‘White-out’, single bubbles cannot be distinguished	Yes

The O’Dive™ recordings were transmitted to the manufacturer as per the intended use of the device and graded offline using Azoth Systems’ proprietary algorithm. VGE assessment was provided for both the right and left subclavian measurements separately. It should be noted that the Azoth System 0–4 scale is not equivalent to VGE Doppler grading on the KM or Spencer scales (but is also ordinal).

### 2.4 Comparison of Venous gas Emboli assessments from different devices

Since both the Butterfly iQ^TM^ and Vivid q^TM^ measurements are graded on the same EB ordinal scale, they can be compared using weighted kappa statistics. Note that this is not the case for the O’Dive^TM^ which uses a different scale. The weighted kappa statistic with linear weights was therefore used to quantify the agreement between the VGE grades obtained with the Butterfly iQ™ and the Vivid q™, as established in prior VGE agreement work (Sawatzky and Nishi, 1991) (Germonpré et al., 2014). Since EB grade data is ordinal, the weighted kappa statistic better accounts for the degree of agreement compared to Cohen’s kappa statistic for which agreement is binary (Cohen, 1960) (Cohen, 1968) (Kraemer, 1980). For example, a disagreement is deemed “worse” between grades 1 and 4 than between grades 1 and 2. The kappa statistic falls between -1 and 1, where 0 corresponds to the value expected by chance and 1 represents perfect agreement. An interpretation of degree of agreement is shown in table 2, adapted from Landis et al. (Landis and Koch, 1977).

**TABLE 2:** Interpretation of the kappa statistic proposed by Landis et al., 1977 (Landis and Koch, 1977).

Below 0.00	Poor
0.00–0.20	Slight
0.21–0.40	Fair
0.41–0.60	Moderate
0.61–0.80	Substantial
0.81–1.00	Almost Perfect

To determine if there was an association between device and image quality obtained, the percentage of times a good quality image was obtained by each device was also calculated. This was done post-hoc as a means to semi-quantify the report from operators in the field that they had more difficulty acquiring good images with the Butterfly iQ™. The z-score test was used to compare the proportions of unusable versus total data (successful collection with at least one device) between the Butterfly iQ™ and Vivid q™. A *p*-value of *p* <0.05 was set a priori as the level of statistical significance.

Additionally, the VGE data derived from each device were binarized based on VGE presence. For this, EB grades of 1-5 were positive and an EB grade of 0 was negative for VGE for the Vivid q™ and Butterfly iQ™. Similarly O’Dive™ ratings of 1-4 were positive while grade 0 was negative. Sensitivity and specificity to VGE for both the O’Dive™ and Butterfly™ were then calculated using Eq.1, 2, as compared to the Vivid q™. For the O’Dive™, values were computed for the left and right subclavian measurements independently, as well as for the highest of the left or right.

*Equation 1*

$$sensitivity=\;\frac{number\;of\;true\;positives}{number\;of\;true\;positives+number\;of\;false\;negatives}$$

*Equation 2*

$$specificity=\;\frac{number\;of\;true\;negatives}{number\;of\;true\;negatives+number\;of\;false\;positives}$$

A Spearman rank-order correlation was also used to measure the strength of the association between VGE assessments obtained from the O’Dive™ (Azoth Systems’ proprietary non-disclosed analysis on 0–4 ordinal scale, again left, right or highest of the two) and of the Vivid q™ (EB grades 0–5 by human raters from echocardiography). Statistical analyses were performed in GraphPad Prism 9 (GraphPad Software, Inc., La Jolla, CA, United States). The Spearman rho coefficient is presented together with its 95% confidence interval. All other results are presented as mean ± standard deviation.

## 3 Results

The ultrasonic assessment of VGE with the newer handheld Butterfly iQ™ and O’Dive™ was compared to that of the standard Vivid q™ often used in diving research. A total of 75 volunteers were enrolled in the study over the course of 21 months. Recordings that were poor quality due to field acquisition difficulties, or where the same volunteer and time point were missing for paired device comparisons, were excluded. A total of 141 matched recordings were compared between the Butterfly iQ™ and Vivid q™, and 173 pairs were compared between the O’Dive™ and Vivid q™.

### 3.1 EB Grade Comparison between the Butterfly iQ™ and Vivid q™

Eftedal-Brubakk VGE grades derived from the Butterfly iQ™ echocardiography data were compared to the Vivid q™ grades at the same time points for each volunteer.

The kappa value for the Vivid q™ and Butterfly iQ™ was calculated from the contingency table shown in Table 3 as 0.52 ± 0.06 (*n* = 141), reflecting moderate agreement.

**TABLE 3:** Contingency table showing Eftedal-Brubakk (EB) grade agreement between the Vivid q™ and Butterfly iQ™. The weighted kappa agreement between EB grades derived from the Butterfly iQ™ and the Vivid q™ measurements was found to be 0.52 ± 0.06.

	Vivid q^TM^ EB grade							
		Grade 0	Grade 1	Grade 2	Grade 3	Grade 4	Grade 5	Totals
**Butterfly iQ^TM^ EB Grade**	**Grade 0**	77	6	4	3	3	0	93
	**Grade 1**	10	6	2	3	1	0	22
	**Grade 2**	1	2	3	5	0	0	11
	**Grade 3**	2	1	1	3	4	0	11
	**Grade 4**	0	0	0	0	2	2	4
	**Grade 5**	0	0	0	0	0	0	0
	**Totals**	90	15	10	14	10	2	141

In terms of the quality of recordings obtained by the Butterfly iQ™ and Vivid q™, 8.8% of the time, a quality image was produced by only the Butterfly iQ™, 17.1% of the time a quality image was obtained by only the Vivid q™, and 74.1% of the time both devices produced good quality recordings. The difference in proportions of unusable vs. total data acquired was significant between Butterfly iQ™ and Vivid q™ (*p* = 0.016).

### 3.2 Sensitivity and Specificity of Handheld devices to the Presence of Venous gas Emboli

The Vivid q™ derived grades were binarized based on the presence, or absence, of VGE, and used as ground truth to calculate sensitivity and specificity of the Butterfly™ and Vivid q™. The agreement between the Butterfly iQ™ and the Vivid q™ is shown in contingency Table 4 which enumerate the true positives, false positives, true negatives and false negatives. The sensitivity and specificity of the Butterfly iQ™ were 68.6 and 85.6% respectively (*n* = 141).

**TABLE 4:** Contingency table showing venous gas emboli (VGE) presence agreement between the Butterfly iQ^TM^ and Vivid q^TM^. The Butterfly iQ™ sensitivity to VGE was found to be 68.6% and its specificity 85.6%.

	Vivid q^TM^			
**Butterfly iQ^TM^**		VGE	No VGE	Total
	**VGE**	35	13	48
	**No VGE**	16	77	93
	**Total**	51	90	141

Similarly, the agreement between the O’Dive™ to the Vivid q™ is shown in contingency Tables 5, 6, 7 for the left, right and highest of left or right subclavian measurements respectively. The O’Dive™ left and right VGE grades as given by the proprietary Azoth Systems assessment were the same for 136/173 measurements. The O’Dive™ sensitivity to VGE was found to be 26.7% and its specificity 94.9% if using the left subclavian measurements, or 33.3 and 89.8% respectively if using the right subclavian measurements, or 42.7 and 86.7% if using the highest of the right and left measurements (all *n* = 173 pairs respectively).

**TABLE 5:** Contingency table showing venous gas emboli (VGE) presence agreement between the Vivid q^TM^ and the O’Dive™ left measurement. The O’Dive™ left measurement sensitivity was found to be 26.7% and its specificity 94.9%.

	Vivid q^TM^			
**O’Dive^TM^ left**		VGE	No VGE	Total
	**VGE**	20	5	25
	**No VGE**	55	93	148
	**Total**	75	98	173

**TABLE 6:** Contingency table showing venous gas emboli (VGE) presence agreement between the Vivid q^TM^ and the O’Dive^TM^ right measurement. The O’Dive™ right measurement sensitivity was found to be 33.3% and its specificity 89.8%.

	Vivid q^TM^			
**O’Dive^TM^ right**		VGE	No VGE	Total
	**VGE**	25	10	35
	**No VGE**	50	88	138
	**Total**	75	98	173

**TABLE 7:** Contingency table showing venous gas emboli (VGE) presence agreement between the Vivid q^TM^ and the highest of the two O’Dive^TM^ measurements (left or right). The O’Dive™ sensitivity was found to be 42.7% and its specificity 86.7% if using the highest of the left and right subclavian measurements.

	Vivid q^TM^			
**O’Dive^TM^ highest**		VGE	No VGE	Total
	**VGE**	32	13	45
	**No VGE**	43	85	128
	**Total**	75	98	173

The Spearman rho coefficient between O’Dive™ VGE ordinal ratings and the EB grades from Vivid q™ assessment was 0.36 [95% CI: 0.21–0.48] (*p* <0.0001, *n* = 173 pairs) using the left subclavian O’Dive™ measurements, showing a weak but statistically significant correlation. Similarly, the correlation coefficient was exactly the same using the right subclavian measurements: 0.36 [95% CI: 0.21–0.48] (*p* <0.0001, *n* = 173 pairs). Finally, the correlation coefficient was 0.39 [95% CI: 0.26–0.52] (*p* <0.0001, *n* = 173 pairs).

## 4 Discussion

In the last twenty years, POCUS has seen wide interest and increased adoption in a variety of clinical and paramedical settings (Smallwood and Dachsel, 2018). New hand-held devices have for example been evaluated in obstetric anesthesiology (Gonzalez Fiol et al., 2020) and cases relating to the termination of resuscitation during cardiac arrest (Reynolds and Del Rios, 2020). In environmental physiology research, including diving, experiments are often conducted in the field with limited resources in austere environments, making POCUS particularly attractive. In DCS research where ultrasound is often used for VGE quantification, it may be beneficial to use a more portable device if it can reliably assess VGE. In this study, we collected a total of 141 Butterfly iQ™ and 173 O’Dive™ measurements to be compared with standard echocardiography based on the Vivid q™. Measures were taken to minimize the time between Vivid q™ acquisition to the evaluated devices, to account for the fact that VGE evolution post-dive can change over time. However, a practical limitation is that lag times could still be up to 5 min between measurements to allow for correct positioning and saving of recordings. O’Dive™ grading was performed by the manufacturer’s proprietary algorithm as per the expected commercial use of the device by divers. Echocardiography recordings from both the Vivid q™ and the Butterfly iQ™ were graded by two independent raters and validated by an additional two experienced raters in case of disagreement, to minimize inter-rater variability in EB grading.

The sensitivity and specificity of the Butterfly iQ™ to VGE were 68.6 and 85.6% respectively, as compared to the Vivid q™ assessment. The overall weighted kappa agreement between the two was only 0.52 ± 0.06 reflecting moderate agreement. The ability of the Butterfly iQ™ to provide effective cardiac imaging has previously been evaluated for clinical practice. It was found particularly effective for emergency use in under-resourced communities, and/or for patients at high cardiac risk (Mchechesi et al., 2021). The cardiac POCUS curriculum now includes the Butterfly iQ™ for some medical students (Jujo et al., 2021). Two documented disadvantages are that the devices are prone to overheating and the need for internet-based cloud (Burleson et al., 2020).

**FIGURE 3:**
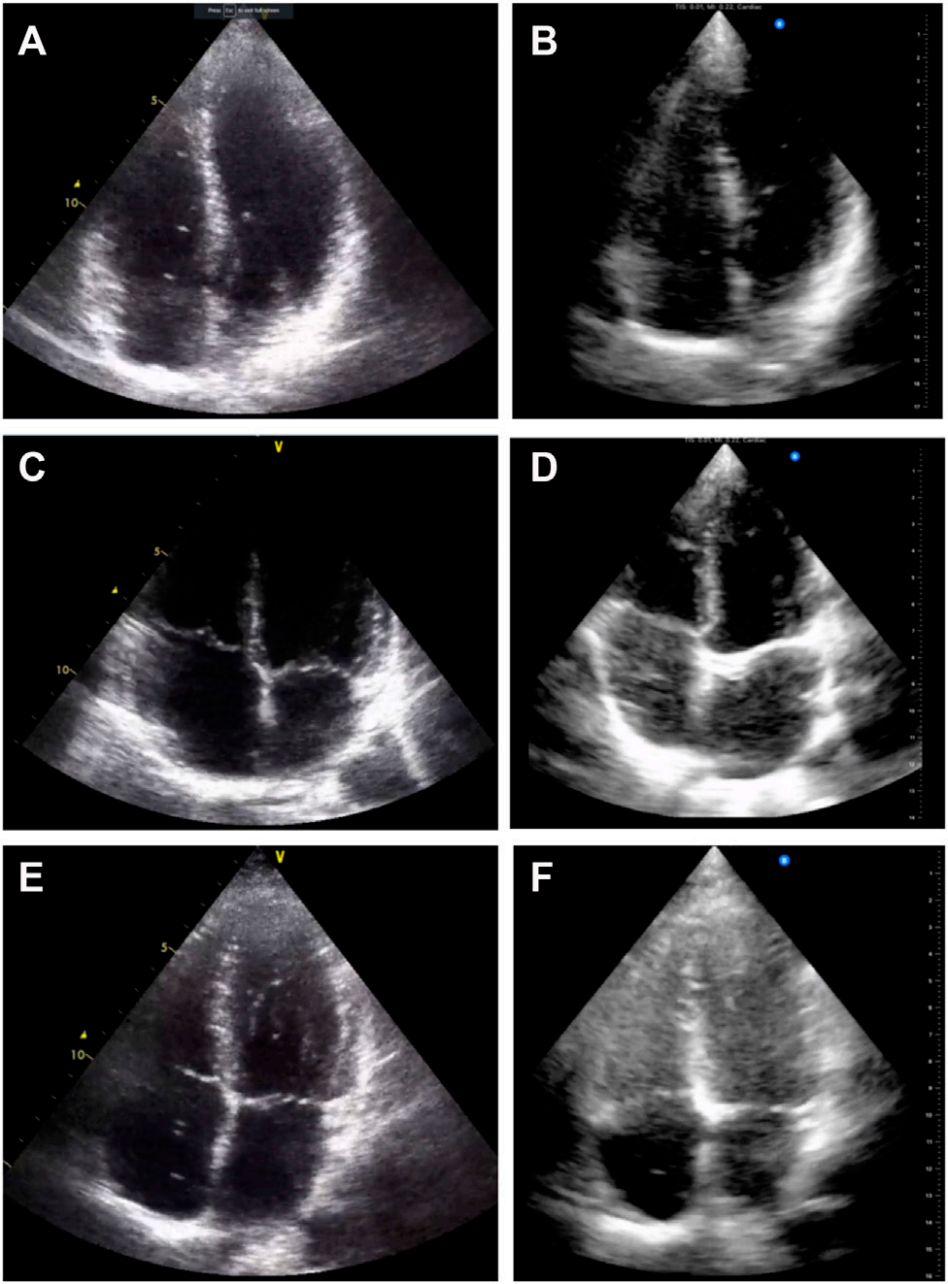
Example echocardiography images acquired with the Vivid q™ **(A)**, **(C)**, and **(E)** and with the Butterfly iQ™ **(B)**, **(D)**, and **(F)**. Three different volunteers at the same measurement time point are presented for comparison **(A–B)**, **(C–D)**, and **(E–F)**.

In practice, the Butterfly iQ™ was able to produce images that were graded similarly for when the quality of the video was good. We found a significant decrease in the proportion of usable data with the Butterfly iQ™ in our study compared to the Vivid q™. Representative examples comparing the images obtained from different volunteers with both devices are shown in Figure 3. It is noticeable that in select cases image contrast in different regions is less ideal with the Butterfly iQ™. One reason may be that gain with the device is adjusted as a global variable and cannot be tuned separately for different depths of the image as is the case for standard clinical systems. Additionally, the footprint of the probe is larger than normal phase-array transducers. This has been noted by others as impeding good intercostal positioning for cardiac imaging (Burleson et al., 2020). The noted decrease in image quality was likely compounded by us having technicians trained for research purposes and not clinically (non-cardiologists/sonographers) acquiring the data. In practice, this acquisition by less trained personnel is not unusual of many diving research studies. Portable device evaluation could therefore include ease of use with minimal training.

The O’Dive™ showed sensitivity to VGE between 26.7% (left subclavian) and 42.7% (highest subclavian) compared to the Vivid q™. Similarly, specificity was between 86.7% (highest subclavian) and 94.9% (left). We found an overall weak positive correlation between the O’Dive™ 0–4 proprietary ordinal assessment scale and the EB grades derived from echocardiography with the Vivid q™ (Spearman r = 0.36 with either left or right subclavian measurements, or r = 0.39 where the highest of the two subclavian measurements were used for analysis, all *p* <0.0001).

Some differences in quantification of VGE between echocardiography and Doppler audio measurements are to be expected. First, the detection of VGE is based on different principles. In Doppler ultrasound, VGE are detected based on their velocity in blood, specifically in the subclavian vein or precordium. VGE from Doppler and imaging have been correlated to DCS risk in the past (Gardette, 1979) (Vann et al., 1982) (Eatock, 1984) and it is accepted that absence of VGE is a good indicator of low DCS risk (Doolette, 2016). A recent meta-analysis looking more specifically at subclavian recordings found that if dive severity is taken into account, then subclavian Doppler is a better predictor of DCS compared to precordial site monitoring (Hugon et al., 2018). This is of interest for the development of devices such as the O’Dive™ that may be easy for divers to position on themselves reliably and leverage these results to reflect time-depth exposure. In echocardiography, VGE are seen as moving bright spots against the dark background of the venous atrium and ventricle. One prior study suggested that precordial Doppler and echocardiography yield similar VGE grading results (Blogg et al., 2010). Another study, however, found higher sensitivity to VGE with pulsed Doppler in the outflow area of the right ventricle 1–2 cm below the pulmonary valve in the heart compared to multiple views in 2D echocardiography (Boussuges et al., 1998). In the latter, the authors noted a contributing factor being lesser image quality in some volunteers on imaging. Both of these prior studies focused on comparing Doppler acquired in the heart region with echocardiography. Since the O’Dive™ measures the subclavian vein, additional discrepancies to echocardiography could be expected due to the different anatomical locations measured. Due to the different principles for VGE detection in Doppler and echocardiography, as well as different anatomical sites measured, it is therefore important to note that our study, by design, cannot validate the O’Dive per se, but only assess its degree of agreement in VGE quantification with the Vivid Q™.

A previous study used the O’Dive^TM^ in conjunction with echocardiography (Balestra et al., 2022) and found that high-grade VGE were observed after deep closed-circuit rebreather diving. The authors also performed a Spearman rank order correlation between the VGE assessed with the O’Dive^TM^ and EB grades from echocardiography, and found r = 0.81 (with *n* = 7 divers compared). The study also reported good agreement after binarizing the assessment scales used to low bubble grade (LBG) defined as 0–2 for O’Dive™ proprietary assessment and 0–2 for EB, and high bubble grade (HBG) defined as 3-4 for O’Dive™ and 3-5 for EB. A notable difference comes from the different type of diving performed. The study from Balestra et al. (2022) focused on a small number of highly technical dives using rebreathers on trimix, with an average depth of 97.3 m, compared to mostly open circuit no-decompression dives in our study. The authors noted that these dives resulted in a high incidence of high VGE grades, in contrast to our study. Since LBG comprises 0 but is not limited to it, this may explain the differences observed in our study, especially given the lower overall EB grades. Other differences may include environmental or operator-related variability. Our lower correlation coefficient is consistent with the finding that the O’Dive™ measuring in the subclavian vein was less sensitive to VGE circulating in the venous heart chambers as assessed by standard echocardiography.

Currently, neither device tested would be able to replace standard echocardiography for VGE assessment. The O’Dive™ sensitivity to VGE was poor compared to echocardiography, and Butterfly iQ™ only resulted in moderate agreement due to increased acquisition quality difficulties. Portability and ease of use may however be useful in practice for studies where a coarser quantification is acceptable. The specificity of the left O’Dive™ measurement to VGE was high and studies where this metric is of interest could consider using this device, for example in validating new dive procedures/tables where a high proportion of Grade 0 measurements are a desired outcome due to the absence of VGE being a good measure of decompression safety (Pollock, 2007) (Blogg and Møllerløkken, 2012). Further research is also needed to better quantify the impact of acquisition quality on VGE quantification in both echocardiography and Doppler audio recordings.

## 5 Conclusion

There is currently a need for small, portable devices with long battery lives to obtain more VGE data in the field to better understand inter- and intra-subject variability in post-dive VGE in divers. This study compared two new handheld ultrasound devices against the Vivid Q™ device that is currently used to monitor post-dive VGE in divers. We found poor sensitivity to VGE for the O’Dive™ in comparison to the Vivid q™, and an overall weak correlation between the two ordinal assessment scales. This may be due to the different anatomical locations measured. Further studies to validate the O’Dive™ proprietary grading system are warranted. There was only moderate correlation in VGE grades for the Butterfly iQ™ when compared to the Vivid q™, partially due to acquisition difficulties in the field with a larger probe. This means that the Butterfly™ is currently not an adequate replacement for grading quantification of VGE. Nevertheless, specificity to VGE was high and studies where this metric is of interest could consider using this device.

## Data Availability

The raw data supporting the conclusions of this article will be made available by the authors, without undue reservation.
